# Supplementary material for: Ultrafast all-optical coherent control of single silicon vacancy colour centres in diamond
Source: Nat Commun. 2016 Nov 14;7:13512. doi: 10.1038/ncomms13512 (PMC5114561; doi:10.1038/ncomms13512)
Supplement: Supplementary Information — Supplementary Figures 1-5, Supplementary Notes 1-3 and Supplementary References [file ncomms13512-s1.pdf]

Supplementary Figure 1

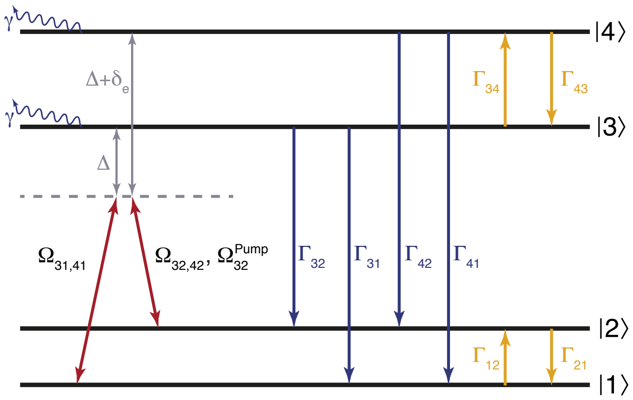

Supplementary Figure 1 – Schematic picture of theoretical model: The picture shows a schematic representation of the theoretical model used to simulate the data presented in the main text. Red arrows indicate driving laser fields which can either resonantly drive the transitions highlighted in Supplementary Fig. 1 of the main text (one-photon experiments) or drive two $mathit\Lambda$-schemes including both ground states and the lower or upper excited state, respectively (two-photon experiments). The detunings of the Raman rotation laser from both $\mathit{\Lambda}$-schemes are then $\mathit{\Delta}$ for the lower scheme and $\mathit{\Delta}+\delta_e$ for the upper scheme, with $\delta_e/2\pi$=259 GHz being the excited state splitting. Spontaneous decay rates $\mathit{\Gamma}_{ij}=\frac{1}{\tau_{ij}}$ between the excited and the ground states are indicated with blue arrows, while phonon-mediated decay and thermalization rates within the ground or excited state manifolds are indicated in yellow. Furthermore, a pure dephasing γ has been included in the model to fit the resonant Ramsey interference experiments. This dephasing γ has been assumed to be equal for both excited states and is most likely caused by residual phonon broadening.

Supplementary Figure 2

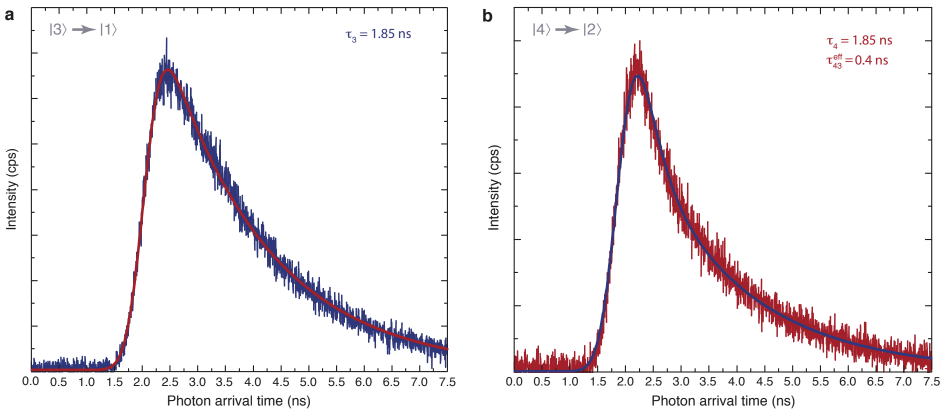

Supplementary Figure 2 – Lifetime measurements of the excited states of the SiV$^-$: Measurements of the arrival times of photons originating from the lower (a) and upper (b) excited state under non-resonant pulsed excitation. The histogram in (a) is fitted using a convolution of a Gaussian APD jitter (τ_j=0.357 ns) and a single exponential decay (red line), revealing a lifetime of state $|3\rangle$ of $\tau_3 = 1.85\,ns$. To model the decay of the upper excited state $|4\rangle$ in (b) a convolution of the Gaussian APD jitter with two exponential decays (blue line) has to be used due to the fast decay from $|4\rangle$ into $|3\rangle$. This reveals a decay of $|4\rangle$ into the ground states with $\tau_4 = 1.85\,ns$ and of $|4\rangle$ into $|3\rangle$ with $\tau_{43}^{\mathrm{eff}} = 0.4\,\mathrm{ns}$.

Supplementary Figure 3

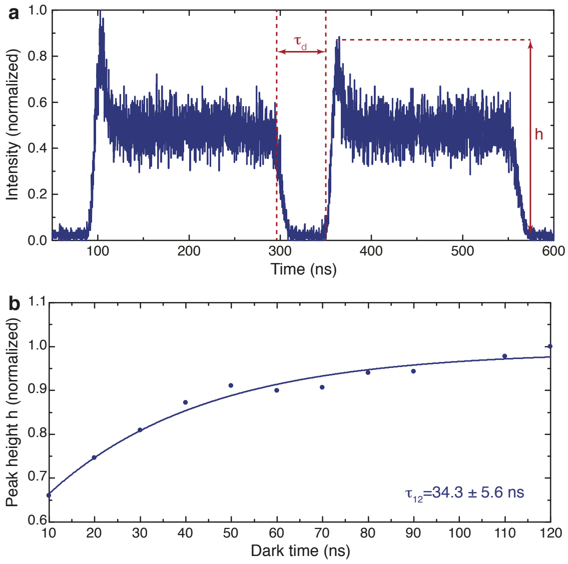

Supplementary Figure 3 – Measurement of T_1^{Orbit} via optical pumping: Resulting fluorescence signal of the pulse sequence consisting of two 200 ns long laser pulses is shown in (a). Both pulses are resonant with transition D. The first pulse pumps the population into the lower ground state while the second pulse probes the retrieval of population in the upper ground state in dependence of the dark time τ_{d} between both pulses. The height of the rising edge of the probe pulse is recorded for several dark times and depicted in (b). An exponential fit of the data results in τ_{12}=34.3±5.6 ns for the thermalization from the lower to the upper ground state.

Supplementary Figure 4

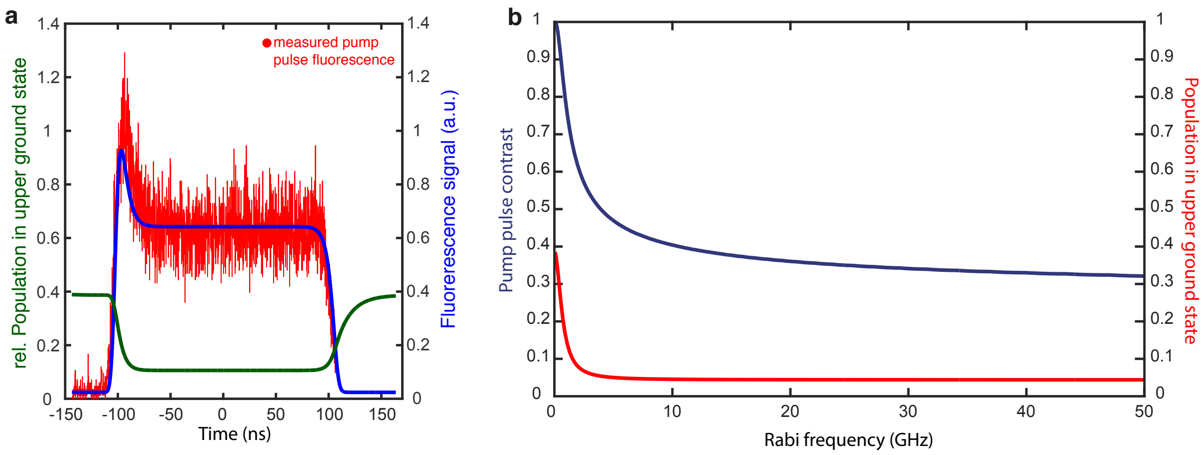

Supplementary Figure 4 – Simulations of optical pumping efficiency: (a) Simulated pump pulse fluorescence (blue, right y-axis) compared with measured pump pulse fluorescence (red) as well as relative population in upper ground state (green, left y-axis). (b) Simulated pump pulse contrast between rising edge peak and equilibrium value at the end of the pulse (blue, y-left axis) as well as population in upper ground state (red, right y-axis) depending on the pump pulse Rabi frequency.

Supplementary Figure 5

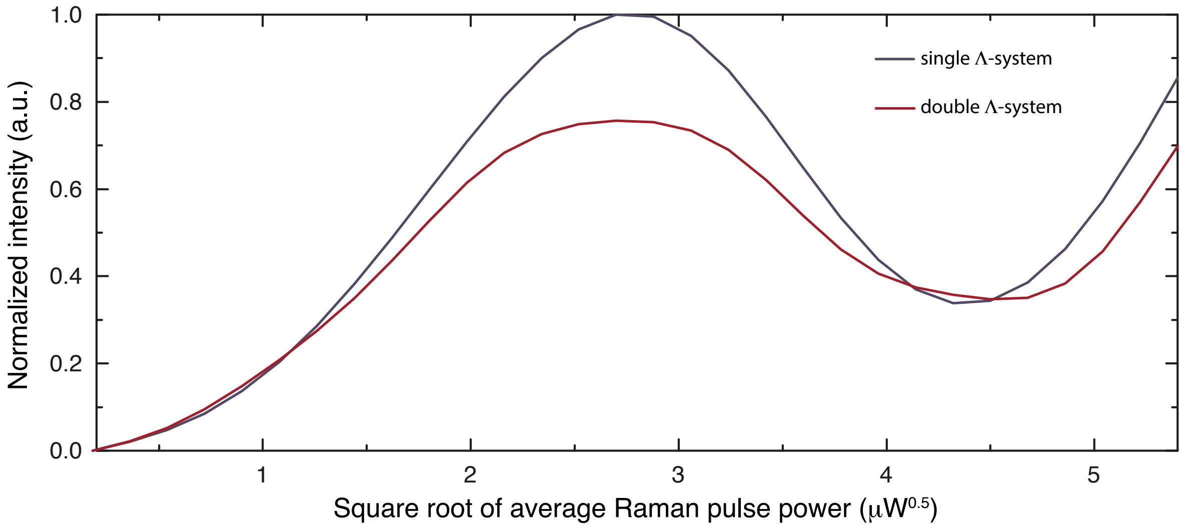

Supplementary Figure 5 – Simulation of Raman-Rabi-oscillations: Simulations of Raman-Rabi-oscillations assuming a single Λ-system consisting of levels $|1\rangle$, $|2\rangle$ and $|3\rangle$ (blue) and a double-Λ-type scheme including the $|1\rangle \leftrightarrow |2\rangle \leftrightarrow |3\rangle$ and the $|1\rangle \leftrightarrow |2\rangle \leftrightarrow |4\rangle$ subsystems (red).

Supplementary Note 1 - Determination of rates

We model the dynamics of the coherently driven SiV^- centre using a full four-level density matrix formalism with two ground states $|1\rangle$ and $|2\rangle$ as well as the two excited states $|3\rangle$ and $|4\rangle$ as basis states. A schematic representation of the model indicating all important parameters is depicted in Supplementary Fig. 1.

The electronic structure of the SiV^- can be described as a combination of two Λ-type systems between $|1\rangle$, $|2\rangle$ and $|3\rangle$ as well as between $|1\rangle$, $|2\rangle$ and $|4\rangle$, respectively. The Hamiltonian of this four level system[1] in the rotating frame is given by

$$\boldsymbol{H} = \frac{1}{2}\begin{pmatrix} 0 & 0 & -\Omega_{31}(t) & -\Omega_{41}(t) \\ 0 & 0 & -\Omega_{32}(t) - \Omega_{32}^{\mathrm{Pump}}(\mathrm{t}) & -\Omega_{42}(t) \\ -\Omega_{31}(t) & -\Omega_{32}(t) - \Omega_{32}^{\mathrm{Pump}}(\mathrm{t}) & 2\Delta & 0 \\ -\Omega_{41}(t) & -\Omega_{42}(t) & 0 & 2(\Delta+\delta_{\mathrm{e}}) \end{pmatrix} \tag{1}$$

where Δ and $\Delta + \delta_e$ are the detunings of the incident laser pulses on both possible Λ-systems, with Δ=0 GHz for the resonant control experiments and $\Delta/2\pi$=500 GHz for the Raman transfer experiments. The time-dependent Rabi frequencies $\Omega_{ij}(t)$, where i and j stand for the excited and ground states, respectively, have been set up such that they reproduce the pulse shapes and lengths measured in the experiment via autocorrelation and have been linked to the experimental Rabi frequencies by employing a scaling parameter. For the resonant control experiments 12 ps double-exponential-shaped pulses have been created by filtering of the fundamental laser pulses with a Fabry-Perot etalon (Lorentzian pulse shapes in frequency space). For the Raman-based control experiments the fundamental, 1 ps hyperbolic secant-shaped pulses of the Ti:sapphire laser have been used without further filtering. The systems dynamics are then calculated from the time evolution of the density matrix $\boldsymbol{\rho}$, which is given by the master equation:

$$\dot{\boldsymbol{\rho}} = -\mathrm{i}[\boldsymbol{H}, \boldsymbol{\rho}] + \sum \boldsymbol{\mathcal{L}}_{ij} + \sum \boldsymbol{\mathcal{D}}_i \tag{2}$$

Spontaneous emission and thermalization between the states are incorporated into the model using the Lindblad operators

$$\boldsymbol{\mathcal{L}}_{ij} = \Gamma_{ij}(\rho_{ii}\,|j\rangle\,\langle j| - \frac{1}{2}\{|i\rangle\,\langle i|\,,\rho\}) \tag{3}$$

The Γ_{ij} describe the relaxation and thermalization rates from state $|i\rangle$ to $|j\rangle$. We employ the rates Γ_{31}, Γ_{32} and Γ_{41}, Γ_{42} to model the decays from the two excited states $|3\rangle$ and $|4\rangle$ to the two ground states $|1\rangle$ and $|2\rangle$. Additionally, we model decay and thermalization processes within the ground and excited state manifolds. We define the decay rates Γ_{21} and Γ_{43} to model the decay. The corresponding thermalization rates are then given by

$$\Gamma_{12} = \Gamma_{21}\mathrm{e}^{-\delta_g/k_B T} \tag{4}$$

and

$$\Gamma_{34} = \Gamma_{43}\mathrm{e}^{-\delta_e/k_B T} \tag{5}$$

To simulate the resonant Ramsey experiments, an additional excited state dephasing rate $\gamma/2\pi$=160 MHz had to be added to the model with the corresponding operator

$$\boldsymbol{\mathcal{D}}_i = \gamma(\rho_{ii}\,|i\rangle\,\langle i| - \frac{1}{2}\{|i\rangle\,\langle i|\,,\boldsymbol{\rho}\}) \tag{6}$$

This dephasing has been assumed to be equal for both excited and states and most likely results from residual phonon broadening. For the simulations in the main text the master equation is numerically solved and the sum of the two excited state populations, ρ_{33}+ρ_{44}, is plotted because the detection of the phonon side band averages over the population of both states.

For the simulations in this work all spontaneous decay rates have been measured experimentally. To measure the decay rates Γ_{31}, Γ_{32}, Γ_{41}, Γ_{42} from the excited to the ground states as well as the excited state decay and thermalization rates Γ_{43} and Γ_{34}, time correlated single photon counting (TCSPC) measurements are employed. For this purpose the SiV^- is excited non-resonantly using 1 ps long laser pulses at 705 nm. To allow for the detection of spectral lines originating from a specific excited state a Fabry-Perot etalon is added to the detection arm. The fluorescence photons were then detected using an avalanche photodiode (APD) (Excelitas SPCM-AQRH-14) with a jitter of τ_j=0.357 ns and the photon arrival times are recorded using a time tagging module (Picoquant PicoHarp 300). Supplementary Fig. 2a and b show histograms of the photon arrival times recorded for the lower (a) and the upper (b) excited state. To extract the lifetime of the lower excited state $|3\rangle$, the histogram is fitted using a convolution of the Gaussian APD jitter with a single exponential decay. This results in a time constant of $\tau_3 = 1.85\,ns$ for the decay of $|3\rangle$ into both ground states combined. To calculate the decay rates Γ_{31} and Γ_{32}, τ_3 is weighted with the relative strengths $\eta_{\mathrm{C,D}}$

of the two transitions C and D originating from $|3\rangle$. These relative strengths have been determined from the line intensities fluorescence spectrum which have been corrected for different outcoupling efficiencies for both transitions due to different dipole orientations.[2] This results in

$$\Gamma_{31} = \frac{\eta_C}{\eta_C + \eta_D} \cdot \frac{1}{\tau_3} = \frac{1}{1+0.6} \cdot \frac{1}{1.85\,\text{ns}} = 338\,\text{MHz} \tag{7}$$

and

$$\Gamma_{32} = \frac{\eta_D}{\eta_C + \eta_D} \cdot \frac{1}{\tau_3} = \frac{0.6}{1+0.6} \cdot \frac{1}{1.85\,\text{ns}} = 203\,\text{MHz} \tag{8}$$

for the decay rates of state $|3\rangle$ into the ground states. A fit of the histogram of state $|4\rangle$ in Supplementary Fig. 2b using the same fit function does not lead to a satisfying fit as state $|4\rangle$ has an additional, dominant decay channel into $|3\rangle$. Therefore the histogram has to be fitted with a convolution of the APD jitter with two exponential decay functions. The jitter and the decay into the ground states are kept fixed at the values determined for $|3\rangle$ and the second decay constant is used as a free parameter. The resulting fit (blue line) is in good agreement with the measured histogram and the time constant for the decay of $|4\rangle$ into $|3\rangle$ is determined to be τ_{43}^{eff}=0.4 ns. This value is the effective decay time of $|4\rangle$ into $|3\rangle$, including the real decay time of $|4\rangle$ into $|3\rangle$ as well as a small amount of thermalization from $|3\rangle$ back into $|4\rangle$. By taking into account a Boltzmann factor for the thermalization we can therefore estimate the time constant in the excited state manifold as follows:

$$\tau_{43} = \left(\frac{1}{\tau_{43}^{\text{eff}}} - \frac{1}{\exp(\frac{\delta_e}{k_B T}) \cdot \tau_{43}^{\text{eff}}}\right)^{-1} = \left(\frac{1}{0.4\,\text{ns}} - \frac{1}{\exp(\frac{259\,\text{GHz}}{20.83\frac{\text{GHz}}{\text{K}}5\text{K}}) \cdot 0.4\,\text{ns}}\right)^{-1} = 0.436\,\text{ns} \tag{9}$$

This expression leads to

$$\Gamma_{43} = \frac{1}{0.436\,\text{ns}} = 2.29\,\text{GHz} \tag{10}$$

and

$$\Gamma_{34} = \Gamma_{43} \cdot \exp(-\frac{259\,\text{GHz}}{20.83\frac{\text{GHz}}{\text{K}}5\text{K}}) = 0.19\,\text{GHz} \tag{11}$$

Furthermore, by taking the relative strengths $\eta_{A,B}$ of transitions A and B into account, we determine the decay rates of $|4\rangle$ into the ground states to

$$\Gamma_{41} = \frac{\eta_A}{\eta_A + \eta_B} \cdot \frac{1}{\tau_4} = \frac{0.19}{0.19+0.4} \cdot \frac{1}{1.85\,\text{ns}} = 174\,\text{MHz} \tag{12}$$

for transition A and

$$\Gamma_{42} = \frac{\eta_B}{\eta_A + \eta_B} \cdot \frac{1}{\tau_4} = \frac{0.4}{0.19+0.4} \cdot \frac{1}{1.85\,\text{ns}} = 366\,\text{MHz} \tag{13}$$

for transition B. To measure the dynamics within the ground state manifold we employ a resonant optical pumping experiment.[3] We resonantly excite transition D from the upper ground state $|2\rangle$ to the lower excited state $|3\rangle$ using a 200 ns long pulse which leads to a pumping of population into the lower ground state $|1\rangle$. This leads to a decay of the fluorescence from an initially high value to a lower level in equilibrium. This equilibrium value corresponds to a population of 22 % in state $|2\rangle$ and is limited by the ground state thermalization rate Γ_{12}. The same pump sequence is used in the coherent two-photon control experiments. We'd like to note that, for technical reasons, the pump pulses here have been chosen relatively long and the length of pulses can be shortened significantly as the minimum population value in state $|2\rangle$ is reached already after about 40 ns of pumping. Moreover, a slight improvement of initialization fidelity and pumping time can be achieved by carefully controlling the polarization of the pump beam. After a variable dark time τ_d, a second, identical laser pulse is then applied and the revival of the rising edge height h in the fluorescence signal is monitored. Supplementary Fig. 3a shows the fluorescence signal during the pump and readout pulses on transition D. The dependence of h on τ_d allows the calculation of the time constant of the thermalization from $|1\rangle$ into $|2\rangle$, which is also known as T_1^{Orbit} of the ground states. In Supplementary Fig. 3b the rising edge height h of the readout pulse is plotted for various dark times. From a fit using a single exponential function (solid line) we extract τ_{12}=34.3±5.6 ns, which is in good agreement with previously measured values for T_1^{Orbit}.[3] From this we determine the rates within the ground state manifold to be

$$\Gamma_{12} = \frac{1}{34.3\,\text{ns}} = 29.2\,\text{MHz} \tag{14}$$

for the thermalization from $|1\rangle$ into $|2\rangle$ and

$$\Gamma_{21} = \frac{1}{34.3\,\text{ns}} \cdot \exp(\frac{48\,\text{GHz}}{20.83\frac{\text{GHz}}{\text{K}} \cdot 5\,\text{K}}) = 46.3\,\text{MHz} \tag{15}$$

for the relaxation of $|2\rangle$ into $|1\rangle$.

We would like to emphasize that no free parameters have been used to model the resonant (two-level) control experiments in the main text. Furthermore, the same set of parameters is used to describe the Raman-based (three-level) experiments with the ratio of the driving strengths of the two Raman-transitions being the only free-parameter for the simulation of these experiments. Moreover, this ratio can not be set entirely arbitrarily and can only be chosen in a small interval to achieve meaningful simulation results.

Supplementary Note 2 - Optical Pumping

To further investigate the efficiency of the optical pumping sequence and to estimate the limitations it imposes on the fidelities of the Raman-based control scheme we simulated the pump pulse using the above mentioned model. In Supplementary Fig. 4(a) the simulated pump pulse fluorescence signal (blue) is shown in comparison to the measured fluorescence (red). Both signals are in good agreement with deviations mainly originating from distortions of the experimental pump pulse caused by the acousto-optic modulator. Using this pulse we calculate the time evolution of the population of the upper ground state (green), starting from a thermal population of about 40%. This simulation indicates that during the pump pulse a minimum population of about 10.5% is reached, close to the theoretical minimum of about $\frac{\tau_{32}}{\tau_{12}} = \frac{1.85ns}{34.3ns} = 5.5\%$ and limited by the available pump laser power in this experiment. In Supplementary Fig. 4(b) the pump pulse contrast (blue) as well as the population in the upper ground state (red) is displayed for increasing pump Rabi frequencies. While the population rapidly decreases, the contrast of the pump pulse leading edge peak evolves more slowly and levels off at about 40% of the peak count rate. Due to a rise and fall time of the AOM of about 12-14 ns, a delay of 15 ns is used between the shut-off of the AOM and the application of the Raman pulse, to avoid pulse overlaps. The simulation indicates that during this period the population in the upper ground state increases rapidly and reaches a value of 22% when the Raman pulse is applied. Therefore, to improve the initialization fidelity in future experiments, a faster modulator can be used that allows the generation of steeper pump pulses, so that the delay between pump and Raman pulse can be further decreased.

Supplementary Note 3 - Influence of double-Λ-scheme

Due to the doublet ground and excited state of the SiV^- a coherent, Raman-based transfer between the ground states is possible via two seperate three-level Λ-type subsystems involving levels $|1\rangle$, $|2\rangle$, $|3\rangle$ (called Λ_1) and $|1\rangle$, $|2\rangle$, $|4\rangle$ (called Λ_2), respectively. Due to the different relative transition dipole moments, the one-photon-Rabi frequencies of the transitions involved are not identical $((\Omega_A : \Omega_B : \Omega_C : \Omega_D = 0.15 : 0.33 : 1 : 0.44))$. As a further factor the different detunings Δ and $\Delta + \delta_e$ from the excited state levels $|3\rangle$ and $|4\rangle$, respectively, determine the magnitude of the two-photon- (Raman-) Rabi frequencies

$$\Omega_{R1} = \frac{\Omega_C \Omega_D}{2\Delta} \tag{16}$$

and

$$\Omega_{R2} = \frac{\Omega_A \Omega_B}{2(\Delta + \delta_e)}. \tag{17}$$

In consequence the coupling strengths via the two pathways are significantly different. Assuming a detuning of $\Delta = 2\pi \cdot 500GHz$ as used in the experiments, the ratio of the two Raman-Rabi-frequencies Ω_{R1} and Ω_{R1} of Λ_1 and Λ_2 is given by

$$\frac{\Omega_{R1}}{\Omega_{R2}} = \frac{\frac{\Omega_C \Omega_D}{2\Delta}}{\frac{\Omega_A \Omega_B}{2(\Delta+\delta_e)}} = \frac{\frac{1 \cdot 0.44}{2\pi \cdot 500GHz}}{\frac{0.15 \cdot 0.33}{4\pi(500+259GHz)}} \approx 27 \tag{18}$$

According to this simple picture, the transfer through the system Λ_1 should be much more efficient. However, more precisely, the transfers via both pathways do not simply add up but a path interference can be observed depending on the relative phase of both paths caused e.g. by a phase difference $\Delta\phi$ of two separate coupling fields. The full expression of the Raman-Rabi-frequency Ω_R in a double-Λ-system therefore is given by

$$\Omega_R = \sqrt{(\Omega_{R1}^2 + \Omega_{R2}^2 + 2\Omega_{R1}\Omega_{R2} \cdot cos(\Delta\phi))}. \tag{19}$$

A detailed discussion of interference phenomena in multilevel-systems can e.g. be found in.[4] In the experiments presented in this work, a single broadband coupling field is used, i.e. no phase difference should occur between both arms of the Λ-schemes. Furthermore, as the dynamics happen on a very short time-scale (≈1ps), time dependent

phase fluctuations can be neglected and therefore $\Delta\phi \approx 0$ can be assumed. In this limit, no destructive path interference takes place and the the total Raman-Rabi-frequency Ω_R simplifies to

$$\Omega_R \approx \Omega_{R1} + \Omega_{R2}. \tag{20}$$

In Supplementary Fig. 5 a simulated Raman-Rabi oscillation of a single- (blue) and a double-Λ-scheme (red) are shown using pulse parameters and detunings equivalent to the ones used to simulate the experimental data. The Raman-Rabi-frequencies for the two cases only differ very slightly, potentially indicating a small phase difference between the pathways. However, a decrease in oscillation amplitude can be observed for the double-Λ-system. This results from an additional population of the second excited state $|4\rangle$ which in turn leads to additional decoherence. Our model, however, indicates that this is not a result of reduced adiabaticity due to the second Λ-scheme but rather caused by resonant excitation (one-photon Rabi oscillations) due to a small but non-negligible overlap between the broad, detuned rotation pulse and the zero-phonon line transitions. To overcome this limitation a single pulse with smaller bandwidth or two separate narrowband-rotation fields can be used in future experiments to minimize resonant excitation of the SiV^-.

Supplementary References

[1] Brzostowski, B. *et al.* EIT Four-Level Lambda Scheme of Cold Rubidium Atoms. *CMST* **16**, 13–16 (2010).

[2] Hepp, C. *et al.* Electronic Structure of the Silicon Vacancy Color Center in Diamond. *Phys. Rev. Lett.* **112**, 036405 (2014).

[3] Jahnke, K. D. *et al.* Electronphonon processes of the silicon-vacancy centre in diamond. *New Journal of Physics* **17**, 043011 (2015).

[4] Kosachiov, D. V., Matisov, B. G. & Rozhdestvensky, Y. V. Coherent phenomena in multilevel systems with closed interaction contour. *Journal of Physics B: Atomic, Molecular and Optical Physics* **25**, 2473 (1992).
